# Supplementary material for: Time-series transcriptome analysis identified differentially expressed genes in broiler chicken infected with mixed Eimeria species
Source: Front Genet. 2022 Aug 8;13:886781. doi: 10.3389/fgene.2022.886781 (PMC9393255; doi:10.3389/fgene.2022.886781)
Supplement: Supplementary file 2 [file DataSheet1.ZIP › 7dpi_GO.Gsea.1625106495923/GOCC_RESPIRATORY_CHAIN_COMPLEX.html]

Details for gene set GOCC\_RESPIRATORY\_CHAIN\_COMPLEX[GSEA]

|  || Dataset | TMM\_7dpi\_gct\_format\_7dpi\_gct\_format.Class\_4dpi.cls #PC\_versus\_NC.Class\_4dpi.cls #PC\_versus\_NC\_repos |
| Phenotype | Class\_4dpi.cls#PC\_versus\_NC\_repos |
| Upregulated in class | 1 |
| GeneSet | GOCC\_RESPIRATORY\_CHAIN\_COMPLEX |
| Enrichment Score (ES) | 0.8112817 |
| Normalized Enrichment Score (NES) | 3.6588273 |
| Nominal p-value | 0.0 |
| FDR q-value | 0.0 |
| FWER p-Value | 0.0 |
Table: GSEA Results Summary

  

Fig 1: Enrichment plot: GOCC\_RESPIRATORY\_CHAIN\_COMPLEX      
 Profile of the Running ES Score & Positions of GeneSet Members on the Rank Ordered List

  

| SYMBOL | TITLE | RANK IN GENE LIST | RANK METRIC SCORE | RUNNING ES | CORE ENRICHMENT || 1 | COX5A | na | 300 | 0.942 | 0.0073 | Yes |
| 2 | NDUFA2 | na | 303 | 0.935 | 0.0392 | Yes |
| 3 | NDUFB6 | na | 307 | 0.933 | 0.0710 | Yes |
| 4 | NDUFS5 | na | 308 | 0.932 | 0.1030 | Yes |
| 5 | NDUFB7 | na | 323 | 0.915 | 0.1333 | Yes |
| 6 | NDUFB2 | na | 348 | 0.885 | 0.1617 | Yes |
| 7 | NDUFAB1 | na | 356 | 0.876 | 0.1912 | Yes |
| 8 | NDUFA7 | na | 371 | 0.857 | 0.2195 | Yes |
| 9 | NDUFC2 | na | 394 | 0.834 | 0.2463 | Yes |
| 10 | UQCR10 | na | 444 | 0.785 | 0.2692 | Yes |
| 11 | COX6A1 | na | 480 | 0.760 | 0.2923 | Yes |
| 12 | UQCRB | na | 487 | 0.753 | 0.3177 | Yes |
| 13 | NDUFA11 | na | 497 | 0.746 | 0.3426 | Yes |
| 14 | SDHAF4 | na | 516 | 0.730 | 0.3662 | Yes |
| 15 | NDUFA13 | na | 557 | 0.708 | 0.3871 | Yes |
| 16 | NDUFS3 | na | 623 | 0.675 | 0.4049 | Yes |
| 17 | NDUFB3 | na | 656 | 0.657 | 0.4247 | Yes |
| 18 | NDUFS8 | na | 661 | 0.653 | 0.4469 | Yes |
| 19 | NDUFS6 | na | 686 | 0.642 | 0.4669 | Yes |
| 20 | SDHD | na | 708 | 0.632 | 0.4868 | Yes |
| 21 | UQCC3 | na | 735 | 0.620 | 0.5060 | Yes |
| 22 | NDUFA1 | na | 742 | 0.616 | 0.5266 | Yes |
| 23 | UQCRHL | na | 788 | 0.594 | 0.5433 | Yes |
| 24 | NDUFA5 | na | 794 | 0.593 | 0.5632 | Yes |
| 25 | SDHB | na | 840 | 0.575 | 0.5792 | Yes |
| 26 | NDUFB5 | na | 902 | 0.554 | 0.5932 | Yes |
| 27 | NDUFV3 | na | 988 | 0.527 | 0.6042 | Yes |
| 28 | NDUFB4 | na | 1015 | 0.517 | 0.6197 | Yes |
| 29 | NDUFA12 | na | 1023 | 0.513 | 0.6368 | Yes |
| 30 | COX7B | na | 1055 | 0.501 | 0.6514 | Yes |
| 31 | NDUFA6 | na | 1079 | 0.492 | 0.6664 | Yes |
| 32 | NDUFA8 | na | 1080 | 0.492 | 0.6833 | Yes |
| 33 | UQCRQ | na | 1103 | 0.484 | 0.6981 | Yes |
| 34 | NDUFS2 | na | 1124 | 0.477 | 0.7128 | Yes |
| 35 | COX4I1 | na | 1125 | 0.476 | 0.7292 | Yes |
| 36 | SDHC | na | 1135 | 0.473 | 0.7447 | Yes |
| 37 | NDUFB10 | na | 1215 | 0.450 | 0.7535 | Yes |
| 38 | UQCRFS1 | na | 1263 | 0.437 | 0.7646 | Yes |
| 39 | NDUFV1 | na | 1307 | 0.428 | 0.7757 | Yes |
| 40 | NDUFB8 | na | 1330 | 0.423 | 0.7884 | Yes |
| 41 | NDUFS7 | na | 1371 | 0.412 | 0.7992 | Yes |
| 42 | NDUFV2 | na | 1395 | 0.408 | 0.8113 | Yes |
| 43 | UQCRC2 | na | 1700 | 0.332 | 0.7973 | No |
| 44 | NDUFB9 | na | 1842 | 0.299 | 0.7957 | No |
| 45 | NDUFB1 | na | 1995 | 0.272 | 0.7924 | No |
| 46 | BCS1L | na | 2225 | 0.232 | 0.7812 | No |
| 47 | UQCRC1 | na | 2326 | 0.217 | 0.7803 | No |
| 48 | NDUFA4 | na | 2726 | 0.160 | 0.7524 | No |
| 49 | NDUFA9 | na | 2879 | 0.141 | 0.7445 | No |
| 50 | NDUFA10 | na | 3412 | 0.073 | 0.7025 | No |
| 51 | SDHA | na | 3488 | 0.064 | 0.6985 | No |
| 52 | NDUFS1 | na | 3763 | 0.033 | 0.6767 | No |
| 53 | NDUFAF1 | na | 3921 | 0.014 | 0.6640 | No |
| 54 | NDUFS4 | na | 4402 | -0.040 | 0.6252 | No |
| 55 | COX7C | na | 4881 | -0.090 | 0.5884 | No |
| 56 | FOXRED1 | na | 6248 | -0.229 | 0.4820 | No |
Table: GSEA details [plain text format]

  

Fig 2: GOCC\_RESPIRATORY\_CHAIN\_COMPLEX      
 Blue-Pink O' Gram in the Space of the Analyzed GeneSet

  

Fig 3: GOCC\_RESPIRATORY\_CHAIN\_COMPLEX: Random ES distribution      
 Gene set null distribution of ES for **GOCC\_RESPIRATORY\_CHAIN\_COMPLEX**
